# Osteitis Pubis in Athletes in the Era of Pubic-Related Groin Pain: Contemporary Concepts in Diagnosis, Rehabilitation, and Surgical Decision-Making

**DOI:** 10.3390/jcm15187187

**Published:** 2026-09-16

**Authors:** Alessandro Massè, Riccardo Giai Via, Salvatore Risitano, Matteo Giachino

**Affiliations:** 1 Department of Surgical Sciences, Orthopaedic and Traumatology Surgery, University of Turin, Via Giuseppe Verdi 8, 10124 Turin, Italy; 2 Department of Orthopaedic Surgery, Centro Traumatologico Ortopedico (CTO), Via Gianfranco Zuretti 29, 10126 Turin, Italy; 3 Department of Orthopaedic and Traumatology Surgery, ASL Torino 5, Piazza Silvio Pellico 1, 10023 Chieri, Italy

**Keywords:** osteitis pubis, pubic-related groin pain, athlete, rehabilitation, magnetic resonance imaging, injection, pubic symphysis, return to sport

## Abstract

**Background**: Osteitis pubis (OP) is a recognized contributor of anterior pelvic and groin pain, particularly in athletic populations, and is currently included within the wider spectrum of pubic-related groin pain. Despite the growing body of literature, clinical management is still challenging and controversial. Significant conceptual confusion persists among related clinical entities; few reviews address the real-world clinical decision-making process; and a poorly defined “grey area” exists between conservative and surgical treatment strategies. **Objectives**: This extensive narrative review aimed to clarify key unresolved issues in the management of osteitis pubis by examining the available evidence to: (1) distinguish osteitis pubis from other causes of pubic-related groin pain; (2) evaluate the clinical criteria guiding progression through conservative treatment strategies; and (3) define the role and limitations of intermediate interventions between conservative and surgical management. **Methods**: An extensive narrative search of PubMed/MEDLINE for publications dated through 4 August 2026 was performed. Clinical studies, consensus statements, systematic and narrative reviews, randomized and nonrandomized trials, observational studies, and case series addressing epidemiology, pathophysiology, diagnosis, imaging, conservative treatment, intermediate interventions, surgery, and return to sport were considered. Reference lists of relevant articles were also screened to identify additional studies. Eligibility criteria, evidence directness, and design-specific methodological limitations were described. **Results**: The current literature shows considerable heterogeneity in terminology, diagnostic criteria, and imaging interpretation, with inconsistent clinico-radiological correlation. Pubic-related groin pain is a clinical regional classification, whereas OP is used here as an author-proposed clinico-radiological phenotype. Conservative management remains the first-line treatment; however, uniform criteria for defining treatment progression and failure are lacking, and much treatment evidence is indirect or derived from mixed groin-pain populations. Injection-based and other interventional therapies are widely used as diagnostic and therapeutic tools despite limited and heterogeneous evidence. Surgical treatment is reserved for selected refractory cases, although indications and techniques vary considerably across studies. Recent evidence further stresses the frequent coexistence of adductor, aponeurotic, inguinal, and hip-related abnormalities and the need to identify a clinically concordant pain generator before treatment escalation. **Conclusions**: This review offers a decision-oriented synthesis and proposes an evidence-informed clinical framework. Organizing the available evidence within a coherent diagnostic and therapeutic framework supports individualized clinical decision-making and identifies priorities for future high-quality studies.

## 1. Introduction

Groin pain is a substantial problem in sports that combine acceleration, cutting, kicking, skating, or repeated single-leg loading. In professional football, hip and groin injuries account for a persistent time-loss burden despite modest reductions in incidence, and adductor-related presentations predominate [1]. Within this spectrum, pain centered on the pubic symphysis has traditionally been called osteitis pubis (OP). The expression suggests an inflammatory joint disease. Yet, the clinical syndrome is more heterogeneous: local bone stress, enthesis and aponeurotic injury, adductor dysfunction, altered load transfer, and adjacent hip or inguinal pathology may occur alone or together [2,3,4,5].

The Doha agreement provided a useful clinical taxonomy in which pubic-related groin pain is defined by local tenderness of the pubic symphysis and immediately adjacent bone [2]. This definition does not require a particular imaging finding and allows coexistence with adductor-, iliopsoas-, inguinal-, and hip-related entities. More recent European consensus work has reinforced this examination-led model and warned that imaging abnormalities are common in asymptomatic athletes [6]. The nomenclature is not merely semantic. Calling every marrow signal change or pubic irregularity “osteitis pubis” risks mistaking adaptation for disease, overlooking a coexisting lesion, and selecting treatment for the image rather than the patient.

The literature emphasizes conservative management and individualized, progressive rehabilitation [3]. It also documents the scarcity of comparative studies supporting injections or surgery. In recent years, evidence has expanded in four relevant areas: consensus-based classification; reliability of clinical examination; anatomical and imaging characterization of the pubic–adductor complex; and criteria-based rehabilitation and endoscopic surgical techniques. Nevertheless, the transition from rehabilitation to an intermediate procedure or surgery remains poorly standardized.

The distinct contribution of the present review is the decision-oriented synthesis of the grey area between rehabilitation and surgery. Beyond the authors’ 2019 rehabilitation review [3] and the 2026 viewpoint on pubic-related groin pain [7], it classifies the directness and methodological limitations of treatment evidence, separates target-specific diagnostic and therapeutic injections, compares symphyseal operations according to pelvic-ring stability, and links these elements in an explicitly proposed clinical framework.

The aims of this review are therefore to: (1) distinguish symptomatic OP/pubic-related groin pain from associated or alternative causes of groin pain; (2) distinguish evidence-supported conclusions from author-proposed criteria for progression through nonoperative treatment and return to sport; and (3) clarify the indications, limits, and evidentiary gaps of intermediate and surgical treatments. The intended result is a practical, evidence-informed framework that preserves diagnostic uncertainty when the evidence is weak while still supporting timely clinical decisions. The framework is proposed for clinical use and has not been validated.

## 2. Materials and Methods

### 2.1. Literature Search Strategy

An extensive narrative review was conducted to identify the most relevant evidence concerning osteitis pubis and pubic-related groin pain in athletes. PubMed/MEDLINE was searched with a publication-date cutoff of August 2026. The search combined free-text terms for the pubic region and related groin-pain conditions with terms identifying athletes and sport; the complete Boolean strategy and its date limits are provided in Supplementary Material. The reference lists of eligible articles, consensus statements, and previous reviews were manually screened to identify additional pertinent studies. PubMed/MEDLINE was retained as the sole bibliographic database because the objective was a clinical narrative synthesis rather than an exhaustive systematic review. This choice may have missed relevant literature and is acknowledged as a limitation; claims therefore refer to the evidence identified by this search.

### 2.2. Eligibility and Evidence Synthesis

Studies were considered eligible when they addressed at least one clinically relevant aspect of osteitis pubis or pubic-related groin pain, including epidemiology, pathophysiology, terminology, clinical diagnosis, imaging, differential diagnosis, conservative treatment, rehabilitation, injection-based or other intermediate interventions, surgical treatment, complications, or return to sport. Consensus statements, systematic and narrative reviews, randomized and nonrandomized trials, prospective and retrospective observational studies, diagnostic and imaging studies, case series, and technically relevant case reports were considered. Studies concerning non-infectious athletic groin pain were prioritized; reports on infection, inflammatory disease, stress injury, and non-musculoskeletal disorders were retained when relevant to differential diagnosis. Studies of adductor-related, aponeurotic, inguinal-related, hip-related, or mixed athletic groin pain were retained only for differential diagnosis or when they informed a recommendation explicitly labelled as indirect. Predominantly obstetric, postoperative, non-athletic, unrelated hernia, or hip-only populations; conference abstracts; duplicate populations; and reports without extractable pubic-region data were excluded. No language filter was applied during the search. Reports without sufficient English-accessible information for reliable data extraction were excluded, introducing a potential language bias. The complete Boolean search strategy and record-selection summary are reported in Supplementary Material.

Titles and abstracts were assessed for clinical relevance, and potentially eligible full texts were examined. Selection was iterative. Contemporaneous records sufficient to verify the retrieval and intermediate screening counts for the search completed on 4 August 2026 are unavailable; these counts are therefore not reported. The bibliography comprises 57 scientific or clinical sources and one regulatory source (the WADA Prohibited List); these are cited-source counts, not a screened-study inclusion total. Screening and extraction were performed by R.G.V.; uncertain eligibility, population classification, and interpretation were discussed with M.G. and A.M. until consensus was reached. Extracted items included the actual diagnostic population, design, sample size, comparator, follow-up, quantitative outcomes, complications, and evidence directness. Older studies were retained whenever they established diagnostic concepts, imaging findings, rehabilitation principles, injection techniques, or surgical procedures that remain relevant to current practice. Because of substantial heterogeneity in terminology, diagnostic criteria, interventions, and outcome measures, the evidence was synthesized narratively and organized by diagnosis, nonoperative treatment, intermediate interventions, surgical management, and decision-making regarding return to sport.

### 2.3. Evidence Appraisal and Directness

**Figure 1:**
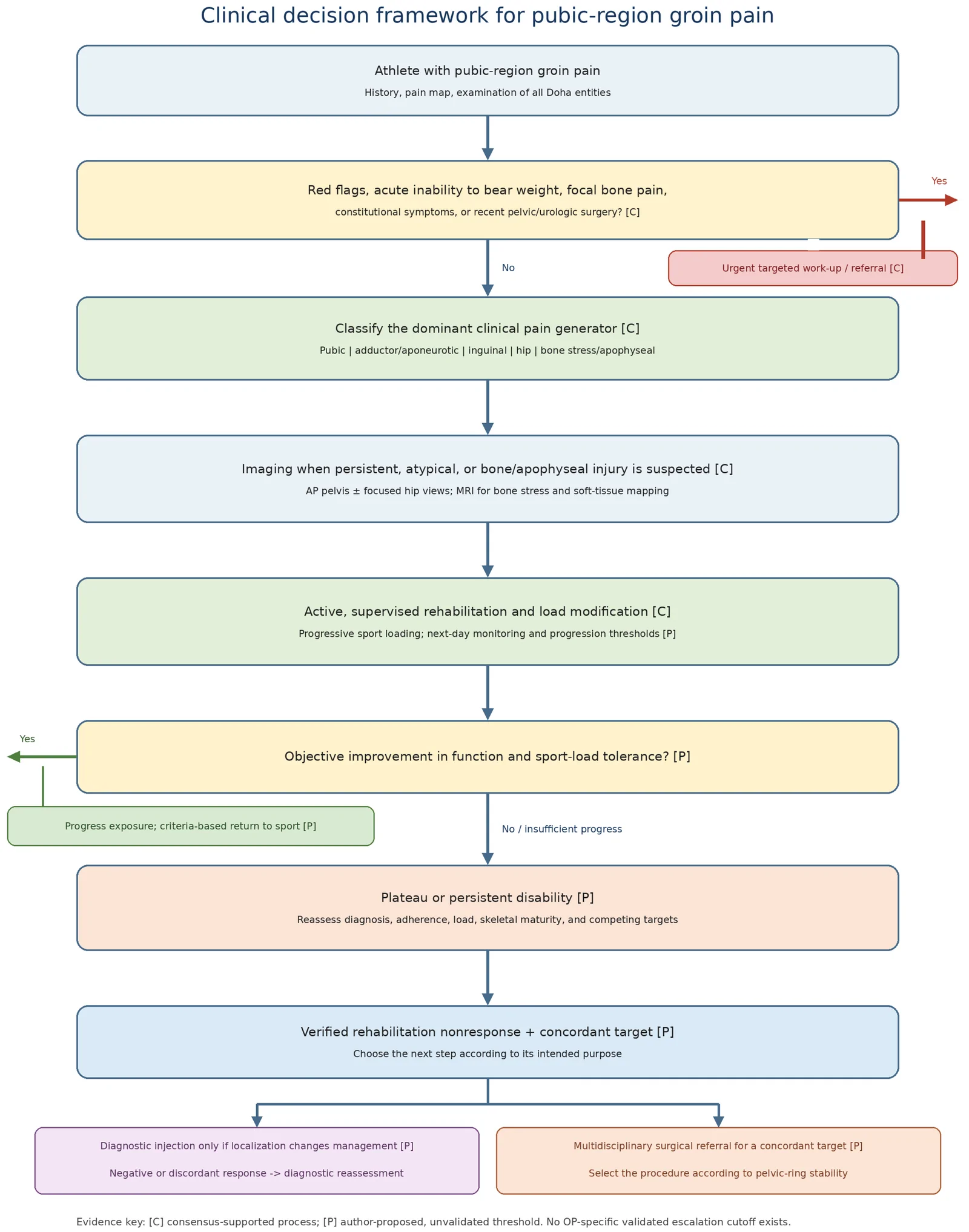
Proposed clinical decision framework for athletes with pubic-region groin pain. A negative or discordant diagnostic injection returns the clinician to reassessment rather than automatically leading to surgery. [C], consensus-supported process; [P], author-proposed unvalidated threshold.

For the principal studies informing clinical recommendations, methodological limitations were appraised with design-appropriate domains: RoB 2 for randomized trials, JBI critical-appraisal domains for observational studies and case series, and AMSTAR 2 domains for systematic reviews. The appraisal was used to describe risk-of-bias concerns rather than to generate a pooled score or exclude studies solely on quality. Evidence directness was classified as direct (a clinically defined pubic-related or osteitis-pubis population), partially direct (a mixed groin-pain population with extractable pubic-specific data), or indirect (an adjacent condition such as adductor-related groin pain). Consensus and expert viewpoints were identified separately and were not treated as primary treatment evidence. Details are reported in Supplementary Table S1. The five-item checklist and Figure 1 were drafted after evidence mapping by R.G.V., reviewed and revised by all three authors through iterative discussion, and approved by author consensus. They were not subjected to a Delphi process, external multidisciplinary review, or prospective validation and are therefore presented explicitly as author-proposed frameworks.

## 3. Terminology: Disease, Clinical Entity, or Imaging Phenotype?

The term OP is used inconsistently. In some reports, it describes pain and tenderness at the symphysis; in others, it denotes pubic bone marrow edema, erosions, sclerosis, joint-space irregularity, or a combination of clinical and imaging findings. Radiology literature has also used “athletic pubalgia”, “sports hernia”, “core muscle injury”, “secondary cleft”, and “prepubic aponeurotic complex injury” for overlapping patterns [4,5]. These labels imply different anatomical targets and can lead clinicians to assume greater diagnostic specificity than the evidence permits.

These terms should be operationally separated. Pubic-related groin pain is a clinical regional classification defined by recognizable tenderness of the pubic symphysis and adjacent bone [2]. Adductor-related groin pain requires adductor tenderness and pain on resisted adduction. Pubic bone stress injury denotes a bone-loading continuum that may include focal marrow edema or fracture; pubic apophysitis is a traction-related injury of an unfused pubic apophysis; and rectus abdominis–adductor aponeurotic injury is a specific soft-tissue lesion that should be reported anatomically [8,9,10,11].

A practical method is to separate three levels of description. First, pubic-related groin pain is a clinical entity: pressure over the pubic symphysis and adjacent bone reproduces the athlete’s recognizable pain [2,6]. Second, the authors propose that OP may be retained as a clinico-radiological phenotype when symptoms and examination are concordant with imaging evidence of pubic bone or symphyseal stress. This is the authors’ conceptual model rather than an established diagnostic standard. Third, individual MRI findings—marrow edema, a cleft sign, aponeurotic injury, adductor lesion, or inferior pubic ligament abnormality—should be reported anatomically rather than treated as synonyms for the syndrome. This hierarchy helps prevent circular reasoning in which an MRI label becomes both the diagnosis and the justification for intervention (see Table 1).

Clinical classification is reproducible only to a limited degree. In athletes with longstanding groin pain, inter-examiner agreement was moderate for adductor-, inguinal-, and iliopsoas-related entities but slight for pubic-related pain (κ approximately 0.12) [12]. A subsequent study found variable reliability for pubic palpation and adductor tests, with examiner experience and standardized instructions influencing agreement [13]. These outcomes do not invalidate the classification; they show that a single positive test should not be overinterpreted. Reproduction of familiar pain, clustering of findings, examination of coexisting entities, and reassessment after load modification seem more defensible than reliance on one maneuver.

## 4. Anatomy and Pathomechanics

The pubic symphysis is a fibrocartilaginous joint within the anterior pelvic ring. Although its physiological motion is small, it transfers forces between the trunk and lower limbs. The rectus abdominis, adductor longus and brevis, gracilis, abdominal fasciae, anterior pubic ligament, and other capsuloligamentous structures interconnect over a compact area. The older “rectus–adductor aponeurosis” model remains clinically helpful but is anatomically incomplete.

A 2022 systematic review emphasized variability in descriptions of the musculotendinous and ligamentous attachments at the symphysis [14]. Cadaveric work published in 2024 found frequent conjoint attachments between adductor longus, adductor brevis, and gracilis, and demonstrated broad connections of these tendons with the anterior and inferior pubic ligamentous structures [15]. These data support the concept of a prepubic aponeurotic complex instead of isolated, independent tendon insertions. However, cadaveric proportions frequently obtained from older donors should not be directly converted into prevalence estimates in young athletes.

Repeated kicking and cutting produce alternating shear, distraction, and compression across this complex. Symptoms may follow an abrupt load spike or accumulate during periods in which sport demand exceeds tissue capacity. Hip motion restrictions, reduced lumbopelvic control, impaired force transfer, prior groin injury, and delayed adductor neuromuscular response may alter load distribution, although causation is difficult to establish. A 2023 study found delayed adductor and abductor muscle reaction in footballers with OP despite no clear between-group difference in maximal strength, suggesting that timing and coordination may matter in addition to peak force [16].

Femoroacetabular morphology provides another plausible link. Restricted hip internal rotation or painful impingement may shift rotational demand toward the anterior pelvic ring. MRI signs of athletic pubalgia are frequent in patients with femoroacetabular impingement syndrome (FAIS), especially in male athletes and those with larger alpha angles [17]. Nevertheless, cam morphology is common in asymptomatic athletes, and clinical associations do not establish that correcting cam morphology is required to resolve pubic pain. The hip should be assessed as a potential coexisting pain generator, not as an automatic upstream cause.

Recent anatomical work has highlighted the inferior pubic ligament (IPL). In a 2025 cross-sectional study, MRI evidence of an IPL lesion was present in 33% of athletes with carefully defined isolated pubic-related groin pain and in none of the adductor-related comparison group; trauma was associated with the finding [18]. The result suggests a potentially meaningful post-traumatic subtype, but wide confidence intervals and lack of outcome-based validation preclude using the IPL signal as a stand-alone diagnosis or surgical indication.

Age and sex modify this scheme. In adolescents, an open pubic apophysis and rapid growth can mimic or coexist with OP, and load management should account for skeletal maturity. Female athletes remain markedly underrepresented in most treatment cohorts. In a prospective study of women’s Premier League football, pubic-related injuries had the longest median time loss among groin categories, but case numbers were small [19]. Pelvic-floor, pregnancy-related, gynecological, and sex-specific biomechanical factors require appropriate consideration without assuming that evidence from male footballers transfers directly.

## 5. Clinical Presentation and Differential Diagnosis

Athletes usually report insidious central or paramedian groin pain that worsens with sprinting, acceleration, cutting, kicking, skating, sit-ups, or resisted adduction. Pain may be unilateral despite bilateral imaging findings. Walking, stairs, rolling in bed, or coughing can become painful as irritability increases. A sudden traumatic onset, focal bony pain, progressive night pain, constitutional symptoms, or pain unrelated to loading shall prompt reconsideration of the diagnosis.

History should establish the exact pain map, onset, recent changes in training or competition, surface and footwear, previous hip/groin problems, response to rest and rehabilitation, and the athlete’s current sporting demands. Duration alone is an unreliable indicator of severity. The clinician should identify which tasks provoke symptoms, how long symptoms persist after activity, and whether the athlete can maintain exposure without deterioration. Previous treatment must be audited rather than simply labeled “failed”: exercise content, dosage, supervision, adherence, concurrent sport load, and progression criteria frequently determine whether a trial was adequate.

Examination begins with gait and single-leg control, followed by range of motion and systematic assessment of the Doha entities [2,6]. Palpation should reproduce the athlete’s familiar pain, not purely elicit local sensitivity. Resisted adduction at more than one hip angle, adductor stretch, abdominal resistance, cough/Valsalva, resisted hip flexion, hip-flexor stretch, and hip provocation tests help identify concurrent entities. FADIR is sensitive to hip-related symptoms but not specific for FAIS. The lumbar spine, sacroiliac region, abdomen, inguinal canal, neurologic system, and—when indicated—genitourinary and gynecological sources should be assessed.

Pain location is not equivalent to tissue source. In a 2025 cohort of 132 male athletes with MRI-confirmed OP, 90% also had an adductor lesion. Although OP was bilateral on MRI in most athletes, symptoms were often unilateral, and sidedness agreed much more closely with adductor longus injury than with OP [20]. The retrospective design and imaging-based entry criteria limit generalization, but the finding is clinically important: a symmetrical pubic MRI abnormality should not override a clearly unilateral adductor presentation.

Red flags include fever, elevated inflammatory markers, immunosuppression, recent pelvic fixation surgery or urological surgery, intravenous drug use, severe pain at rest, inability to bear weight, and suspected malignancy or stress fracture. Septic arthritis or osteomyelitis of the pubic symphysis can initially resemble OP and requires urgent laboratory assessment, blood cultures, and targeted imaging. In older adults or nonathletes, abdominal, genitourinary, gynecological, rheumatological, and postoperative causes deserve greater weight in the differential diagnosis.

## 6. Diagnostic Work-Up

### 6.1. Clinical Classification and Outcome Measurement

No single examination test establishes symptomatic OP [21]. A defensible working diagnosis contains four elements: (1) a plausible load-related history; (2) recognizable pain centered on the pubic symphysis; (3) local tenderness that reproduces that pain; and (4) explicit assessment of coexisting or alternative entities. These four elements are a proposed clinical formulation rather than validated diagnostic criteria. The diagnosis should be revised when the clinical course does not match expectations.

### 6.2. Radiography

An anteroposterior pelvis radiograph, with an additional Dunn or lateral hip view when hip pathology is suspected, is a reasonable first-line examination for persistent symptoms or atypical presentations [6]. Radiographs can show joint-space irregularity, sclerosis, cysts, erosions, bone proliferation, asymmetry, prior avulsion, or hip morphology. Also, Flamingo views have been proposed to assess vertical instability by comparing displacement across the symphysis. Flamingo views are weight-bearing anteroposterior pelvic radiographs obtained during single-leg stance. In chronic cases, radiographs may also show bony bridging across the symphysis. However, protocols and thresholds vary, and dynamic imaging rarely determines treatment in isolation. A threshold greater than 2 mm was used in one historical athletic arthrodesis series [22], whereas other chronic-instability literature has used greater than 5 mm [23]. Physiological motion, sex and parity, positioning, pain-limited loading, and measurement technique affect the result; no universally validated athlete-specific cutoff exists. Flamingo views do not define posterior-ring integrity, which requires clinical assessment and, when indicated, CT or MRI.

Exposure-related adaptation is common. In 2025, Nielsen et al. compared symptomatic male ballers with asymptomatic footballers and asymptomatic non-football athletes. Pubic lucency, proliferation, and sclerosis were substantially more common in both football groups than in non-football controls, but did not differ meaningfully between symptomatic and asymptomatic footballers and correlated poorly with pain and disability [24]. Thus, chronic radiographic abnormalities may document loading history rather than the current pain generator.

### 6.3. Magnetic Resonance Imaging

MRI is the most informative second-line modality when symptoms persist, the diagnosis is uncertain, or bone stress, apophyseal, musculotendinous, or intra-articular disease is suspected. A pelvis protocol should include fluid-sensitive sequences in at least two planes and a sufficient field of view to assess the pubic symphysis, adductors, rectus abdominis/prepubic complex, hips, and alternative bony pathology. High-resolution oblique or dedicated sequences may improve visualization of the adductor-aponeurotic complex [25,26]. When focal bone stress injury is suspected, MRI findings should be interpreted with impact pain, load history, skeletal maturity, and systemic risk factors rather than absorbed into a generic OP label [8].

Potential findings include subchondral marrow edema, an irregular or widened symphyseal cleft, erosions, cysts, disc protrusion, a secondary cleft, adductor enthesis or tendon injury, rectus abdominis injury, and IPL abnormality. MRI is valuable because it maps coexisting lesions, not because any single sign is pathognomonic. Marrow edema has been reported in asymptomatic footballers and may reflect training exposure [27,28]. Earlier studies found associations between certain MRI patterns and symptoms or time to return [26,29,30,31], but differences in definitions, sequences, reference standards, and athlete selection limit the applicability of universal thresholds.

A large MRI cohort of 470 athletes with groin pain found multiple patterns and sex-related differences; approximately one quarter had no MRI abnormality [32]. Conversely, the 2025 OP cohort showed very high coexistence of adductor pathology [20]. Together, these observations illustrate both directions of discordance: symptoms can exist without a visible lesion, and visible lesions can exist without being the dominant source. MRI reports should therefore describe anatomy, severity, laterality, and alternative diagnoses while avoiding deterministic language.

### 6.4. Ultrasound, Computed Tomography, and Diagnostic Procedures

Ultrasound is useful for dynamic assessment of the inguinal canal, evaluation of superficial tendons, and image-guided injection. It is operator-dependent and less comprehensive for the pubic bone and deep pelvic structures. Computed tomography (CT) provides high-resolution bone detail and may assist operative planning, but radiation and limited soft-tissue contrast make it a selective rather than routine test [6]. Bone scintigraphy is now rarely required due to its low specificity and the availability of MRI (see Table 2).

An image-guided local anesthetic injection into the symphysis or a targeted adjacent structure can test whether short-term pain reduction changes a specific provocative task. The result is probabilistic: anesthetic spread, placebo effects and the coexistence of lesions limit specificity. A positive response may strengthen concordance before an invasive procedure; a negative response must prompt reassessment, but does not categorically exclude the region.

## 7. Nonoperative Management

### 7.1. Principles and Initial Symptom Control

Active, supervised rehabilitation is the first-line treatment for most athletes with clinically concordant pubic-related pain [3,33,34,35,36,37]. This recommendation is supported directly by small pubic-specific cohorts, indirectly by adductor-related groin-pain evidence, and by consensus-based practice. The goal is not complete unloading of the symphysis. It is to temporarily reduce provocative exposure, restore tolerable force transfer, and rebuild sport-specific capacity. Absolute rest can decrease symptoms while simultaneously reducing tissue capacity and delaying return.

Initial management should identify a load “floor” that the athlete can tolerate without meaningful next-day worsening. High-speed running, long kicking, repeated cutting, deep lateral lunges, or high-volume abdominal work may be reduced. In contrast, low-irritability cycling, pool exercise, upper-body conditioning, or modified field work maintains fitness. Analgesics and short courses of nonsteroidal anti-inflammatory drugs may be considered for symptom control after individual risk assessment, but should not mask worsening load intolerance. Sleep, nutrition, recovery, and communication with coaching staff are part of treatment.

### 7.2. Criteria-Based Rehabilitation

The strongest historical evidence for longstanding adductor-related groin pain favors active training over passive physical therapy [34]. This is indirect evidence for OP. OP-specific evidence remains limited, but a progressive program that combines lumbopelvic control, hip and trunk strength, graded running, change of direction, and kicking is consistent across cohorts and contemporary recommendations [3,33,35,36]. In 14 professional academy players with longstanding pubic-related pain, a six-level criteria-based protocol produced a high return-to-play rate and one recurrence. However, the mean time to return was approximately 135 days, and the uncontrolled cohort was small [33].

A 2024 randomized study in adolescent male footballers compared a modified active program with traditional physiotherapy over 12 weeks. Both approaches improved outcomes; the modified program produced greater gains in strength and function, whereas the traditional group showed a larger pain reduction [38]. The single setting, adolescent population, intervention complexity, and short follow-up limit interpretation. The result supports active rehabilitation but does not establish one universal protocol.

The following progression principles are author-proposed and have not been validated specifically for OP. Progression may be criteria-based rather than governed by fixed weekly stages. Mild, stable pain during exercise may be used pragmatically when mild, stable, and resolved by the following day. Increasing resting pain, deteriorating gait, weakened performance at the same load, or a prolonged post-exercise flare indicate that the dose should be reduced or the diagnosis revisited. Early exercise can include isometric adduction, anti-rotation trunk work, bridges, and controlled bilateral patterns. Subsequent loading should progress through isotonic and eccentric adduction, single-leg strength, multiplanar trunk control, and rate-of-force development. The Copenhagen adduction exercise can be useful, but should be introduced at an appropriate lever arm and volume. No validated pain-monitoring threshold exists, and use of the Copenhagen adduction exercise is extrapolated mainly from adductor-related groin rehabilitation.

The following field progression is an evidence-informed but unvalidated clinical proposal. Field progression begins with linear running at submaximal speed, then acceleration and deceleration, planned direction change, reactive agility, and sport-specific ball or skating tasks. Kicking should progress from short, low-velocity passes to longer, maximal efforts. High-speed exposure should be measured when feasible. An athlete who is pain-free in the clinic but has not tolerated sprinting, cutting, or repeated kicking has not completed rehabilitation. No OP-specific number of exposures or workload cutoff has been validated.

### 7.3. Extracorporeal Shockwave Therapy and Adjuncts

In Schöberl’s et al. study, 44 footballers were randomized to rehabilitation plus focused extracorporeal shockwave therapy (ESWT; n = 26) or the same program plus sham treatment (n = 18) [35]. The separate rest-based group (n = 51) was not part of that randomized comparison and should not be interpreted as a third randomized arm. A 2024 systematic review of ESWT in athletic populations found that the combined use of ESWT and exercise was promising for OP, but noted heterogeneous protocols and generally limited study quality [37]. ESWT may therefore be offered as an adjunct when progress is slow, with the expectation that it will facilitate rather than replace loading.

Manual therapy, mobility work, and soft-tissue techniques may reduce symptoms or address an individual restriction, but their value is mainly supportive. Passive modalities should not dominate a program. Correction of every asymmetry is neither feasible nor evidence-based; exercise selection should be tied to the athlete’s deficits and sporting tasks.

### 7.4. Defining Progress, Nonresponse, and Return to Sport

“Failure of conservative treatment” is often reported as elapsed time—commonly three to six months—without documenting treatment quality. The authors propose that a more meaningful definition should include: diagnostic reassessment; an adequately supervised, adherent, active program; appropriate control of concurrent sport load; progression into running and sport-specific tasks when possible; and persistent function-limiting symptoms despite modification of identified deficits. Failure to improve despite continued maximal competition or only passive treatment is not equivalent to failure of rehabilitation. This is an evidence-informed clinical proposal, not a validated definition or time threshold.

The Copenhagen Hip and Groin Outcome Score (HAGOS) is a validated patient-reported measure for longstanding hip and/or groin pain with six domains: pain, symptoms, physical function in daily living, physical function in sport and recreation, participation in physical activities, and hip- and/or groin-related quality of life [39]. Serial domain scores can document perceived recovery during rehabilitation. No OP-specific HAGOS cutoff has been validated for treatment failure or return to sport.

Return to sport is a continuum rather than a single clearance event. The following unvalidated, author-proposed domains include: minimal or no tenderness; no clinically relevant next-day response after training; near-symmetrical adductor strength and acceptable force at multiple hip angles; pain-free multidirectional trunk and single-leg tasks; repeated-sprint, acceleration/deceleration, change-of-direction, and kicking or skating exposure at match-relevant intensity; and athlete confidence. No OP-specific validated cutoff exists. HAGOS and sport-specific workload should therefore be interpreted alongside examination and performance. Persistent MRI abnormalities should not independently prevent return when symptoms, function, and load tolerance have recovered, except when a true bone stress injury requires imaging-informed protection.

### 7.5. Adolescents, Pubic Apophysitis, and Bone Stress Injury

Adolescents and young adults with an open pubic apophysis should not be placed automatically on the adult OP pathway. Pubic apophysitis is a traction-related stress injury of the unfused secondary ossification center and may present as adductor-region pain; focal apophyseal widening, asymmetry, cyst-like change, or edema must be interpreted with skeletal maturity and symptoms [9]. A 2026 comparison also found pubic apophyseal MRI abnormalities in asymptomatic elite youth players, including subapophyseal edema in 77.5% versus 18.8% of non-athlete controls, reinforcing that imaging remains non-diagnostic without clinical concordance [10].

When a pubic bone stress injury or fracture is suspected, the immediate priority is protection from painful impact loading, assessment of training-load error and systemic contributors such as low energy availability, and MRI-based definition of injury extent [8]. Progression is symptom- and function-led; high-impact sport is reintroduced only after pain-free daily loading and graded running. Pubic apophysitis and pubic bone stress injury therefore require a bone-healing and maturation framework rather than the same symptom-permissive loading assumptions used for chronic adult pubic-related groin pain.

## 8. Intermediate Interventions: The Clinical “Grey Zone”

Intermediate procedures are frequently used when rehabilitation plateaus, but the athlete or clinician is not ready to consider surgery. Their purpose should be explicit: diagnostic clarification, short-term symptom reduction to enable loading, or treatment of a defined associated lesion. They should not be offered simply because the MRI is abnormal. Diagnostic local-anesthetic injection, therapeutic intra-symphyseal injection, adductor enthesis/peritendinous injection, and prepubic aponeurotic injection have different purposes and should not be treated as interchangeable.

### 8.1. Diagnostic and Therapeutic Injections by Purpose and Target

A diagnostic procedure uses local anesthetic alone, or as a separately interpretable first phase, to test whether a preselected provocative task changes during the expected anesthetic window. The exact target, guidance method, agent, volume, task response, and intended consequence should be recorded. Spread, placebo effects, coexisting lesions, and the absence of a validated response threshold mean that a positive response supports but does not prove localization; a negative or discordant response should prompt diagnostic reassessment rather than automatic progression to surgery [40,41].

#### 8.1.1. Intra-Symphyseal Injection

An intra-symphyseal procedure should be performed with ultrasound or fluoroscopic guidance. Local anesthetic alone can be used for diagnostic localization, whereas corticosteroid may be added when short-term therapeutic symptom modulation is intended. Spread and placebo response limit specificity; target-specific risks include infection, bleeding, post-injection flare, skin or fat atrophy, systemic steroid effects, and unpredictable durability.

Pubic symphysis corticosteroid injection has been described for decades, but evidence is derived mainly from small series with variable technique, chronicity, and rehabilitation [40,42,43]. In a 2011 systematic review, approximately 59% of injected athletes returned to sport, and roughly one-fifth did not respond; however, all included treatment studies were low-level, and the pooled estimates were vulnerable to selection and reporting bias [42]. Earlier series suggested that image guidance and shorter symptom duration might improve response, but neither a standard drug/dose nor a validated response threshold has emerged [40,43].

An injection may be reasonable when pubic pain is clinically and radiologically concordant, symptoms impede participation in rehabilitation, and infection has been excluded. The athlete should understand that the benefit may be temporary and that immediate analgesia does not prove durable disease modification. Repeated corticosteroid exposure near tendon and aponeurotic structures should be approached cautiously. Tendon weakening and rupture are recognized in the general musculoskeletal corticosteroid literature [44], particularly with intratendinous exposure, but no pubic-symphysis-specific complication rate is available. Anti-doping regulations are time-sensitive and the current Prohibited List, washout periods, and any Therapeutic Use Exemption requirement should be checked at the time of treatment [45].

#### 8.1.2. Adductor Enthesis or Peritendinous Injection

When examination and imaging identify a dominant adductor enthesis or peritendinous lesion, ultrasound guidance allows the injectate to be matched to the intended purpose and helps avoid intratendinous corticosteroid placement. Any benefit should be judged by subsequent loading tolerance rather than immediate analgesia alone. Evidence is largely indirect, and tendon weakening or rupture is a general corticosteroid-related concern rather than a pubic-symphysis-specific complication rate.

#### 8.1.3. Prepubic Aponeurotic Injection

A defined rectus–adductor or prepubic aponeurotic lesion may be targeted under ultrasound or fluoroscopic guidance when symptoms and imaging are concordant. Local anesthetic may assist localization, whereas biological injectates remain investigational. Complex regional anatomy, injectate spread, limited comparative evidence, and proximity to tendon, neurovascular, and inguinal structures restrict diagnostic certainty and therapeutic claims.

### 8.2. Platelet-Rich Plasma, Prolotherapy, and Other Procedures

Platelet-rich plasma (PRP) has biological plausibility for tendon or aponeurotic injury; direct evidence for the pubic symphysis is extremely limited. A 2022 case report described improvement after ultrasound-guided PRP injection into the symphysis [46]. Such a result cannot distinguish treatment effect from natural history, altered loading, rehabilitation, or placebo. In the 2025 retrospective OP cohort, return-to-sport proportions among athletes with chronic adductor longus lesions did not differ significantly among the corticosteroid, PRP, and no-injection groups. Still, treatment allocation was non-random and confounded by indication [20]. The study should not be interpreted as evidence of equivalence.

Dextrose prolotherapy has been reported in an uncontrolled case series of elite athletes with chronic groin pain [47]. Other proposed interventions—including pulsed radiofrequency, botulinum toxin, and regenerative preparations—are supported by isolated reports or extrapolation from adjacent conditions [41]. Heterogeneous injectates, targets, guidance methods, co-interventions, and outcome definitions make comparison impossible. These procedures should be described to patients as evidence-limited and, ideally, delivered within prospective protocols.

### 8.3. An Evidence-Informed Checklist Before Escalation

Based on the direct and indirect evidence summarized in this review [2,3,33,34,35,36,37,40,41,42,43,46,47], the authors propose the following unvalidated five-point checklist before any intermediate intervention:

1. Clinical diagnosis: Is pubic-related pain still the main problem, or is another entity now dominant?
2. Target: Does imaging show a plausible lesion that corresponds to the symptoms and provocative tasks?
3. Rehabilitation: Was treatment active, supervised, progressive, sufficiently dosed, and accompanied by appropriate load control?
4. Purpose: Is the procedure diagnostic, intended to facilitate rehabilitation, or definitive?
5. Next step: Which clinical finding or time point will trigger continuation, reassessment, or surgical referral?

This evidence-informed checklist provides a simple framework for structured decision-making in the clinical “grey zone.” It does not assign validated treatment thresholds.

## 9. Surgical Management

### 9.1. Indications and Preoperative Reasoning

Only a minority of athletes with OP require surgery. Reported thresholds range from 3 to 6 months of nonoperative care, but no duration alone is a validated indication [3,48]. The authors propose multidisciplinary surgical review only when an athlete has persistent function-limiting pain, a reproducible and concordant clinical target, failure of an adequately documented rehabilitation program, and informed acceptance of uncertainty. Diagnostic injection may add information in selected cases. Infection, stress fracture, and non-musculoskeletal causes must be excluded.

The operation must match the proposed pain generator. Procedures directed at the symphysis include open or endoscopic curettage/debridement, partial symphysectomy, wedge resection, and arthrodesis. Associated adductor or prepubic aponeurotic pathology may lead to repair, release, or debridement; inguinal-related disease may require posterior wall repair; and symptomatic FAIS may be treated arthroscopically. Combining procedures can be appropriate when more than one diagnosis is well established, but it obscures which component was necessary and may increase morbidity.

Procedure selection should reflect both the dominant pain generator and pelvic-ring stability. Curettage removes degenerative fibrocartilage or subchondral tissue while limiting disruption of stabilizing structures; partial or endoscopic symphysectomy removes a larger central/anterior portion and may carry greater instability risk if resection is excessive. Wedge resection historically aimed to preserve the anterior and inferior stabilizers [49]. Arthrodesis is a stabilization procedure and is most defensible for documented symptomatic instability or salvage after failed resection, not for an imaging abnormality alone [22,23].

### 9.2. Evidence for Pubic Symphysis Procedures

A 2022 review identified only 51 surgically treated athletes across eight studies published from 2000 to 2021, with substantial variation in selection, technique, and outcomes [48]. A broader systematic review included 47 studies and 2737 patients across inguinal-, adductor-, and pubic-related operations, but only 30 athletes underwent an isolated pubic symphysis procedure: 7 arthrodeses and 23 curettage procedures [50]. Return to the preinjury level was reported in 7/7 after arthrodesis and 16/23 after curettage (23/30 overall). These procedure-specific denominators should not be conflated with the much larger overall review population.

Open curettage has produced successful return in selected athletes, but persistent pain, instability, and later fusion have been reported [22,51]. Arthrodesis may stabilize a painful, unstable, or previously resected symphysis but carries risks of nonunion, hardware symptoms, altered pelvic mechanics, and a longer recovery. Wedge resection can reduce the pathological joint while attempting to retain stability; results remain based on small series. Injury to the bladder, vascular structures, spermatic cord, or pelvic ring is uncommon but potentially serious.

Endoscopic curettage and endoscopic symphysectomy should be distinguished according to the extent and location of tissue resection. Limited curettage aims to remove pathological fibrocartilage or subchondral tissue while preserving the stabilizing posteriori structures, whereas symphysectomy involves a more extensive central or anterior resection and may carry a greater risk of postoperative instability [52,53]. In a 2023 series of 10 competitive male footballers with recalcitrant isolated OP, marked pain and patient-reported outcome improvement were reported at a mean follow-up of approximately 6 years, with return to sport at a mean of 4.6 months; transient scrotal swelling occurred in 4 athletes [54]. In 2026, 14/15 eligible patients had minimum 2-year follow-up after endoscopic symphysectomy; 12 had participated in sport preoperatively, 9/12 returned within 1 year (75%), and all 9 who attempted return succeeded, while 3 did not attempt return [55]. Both studies are uncontrolled level-IV series from selected centers and cannot establish superiority over continued rehabilitation or open surgery.

### 9.3. Coexisting Hip, Adductor, and Inguinal Pathology

In selected soccer players with FAIS and pubic marrow edema/OP findings, hip arthroscopy has been associated with improvement regarding symptoms and pubic MRI abnormalities [56]. Endoscopic symphysectomy has also been combined with FAIS surgery in small multicenter series (see Table 3) [57]. These observations support evaluation of the hip but not prophylactic correction of morphology. Hip surgery is justified when the athlete independently meets criteria for symptomatic FAIS and the hip findings are concordant. When hip and pubic procedures are combined, the effect of each component cannot be isolated.

The same principle applies to adductor or inguinal surgery. The high prevalence of adductor lesions in an imaging-selected OP population [20] does not mean that all lesions require release or repair. An adductor procedure needs to address a persistent, clinically dominant lesion after targeted loading has failed. Inguinal repair should be based on an inguinal presentation and surgical assessment, not on the generic term “athletic pubalgia.” The same target-specific principle applies to rectus–adductor aponeurotic procedures. Before symphyseal resection or fusion, the posterior pelvic ring should be assessed clinically and with CT or MRI when indicated; Flamingo-view thresholds vary and cannot select an operation in isolation [22,23].

## 10. Proposed Clinical Decision Framework

Figure 1 integrates red flags, clinical classification, indications for imaging, identification of the dominant pain generator, active rehabilitation, response assessment, the purpose and consequences of diagnostic injection, and criteria for surgical referral. Consensus-supported processes are labelled [C], whereas response thresholds and escalation criteria are labelled [P] as author-proposed and unvalidated.

## 11. Discussion

The evidence identified by this review does not establish a single superior treatment. Historical studies established the main elements that still shape practice: active rehabilitation is preferable to passive care [34]; symphyseal injections provide variable and often temporary benefit [40,42,43]; and surgery is a salvage option for carefully selected refractory athletes [22,48,51]. More recent work has refined, rather than replaced, these principles by supplying a more disciplined way to connect symptoms, examination, imaging, and load. The contemporary model starts with a clinical region—pubic-related groin pain—and then determines whether a pubic bone/symphysis phenotype, an adjacent soft-tissue lesion, or a coexisting entity best explains the athlete’s limitations. The recommendations below distinguish direct pubic-specific evidence from indirect evidence, consensus, and author interpretation.

Three findings illustrate this shift. First, clinical classification is useful but imperfectly reliable [12,13], which argues for standardized examination and longitudinal reassessment. Second, chronic pubic radiographic changes are common in asymptomatic footballers [24], confirming that exposure and symptoms are not interchangeable. Third, MRI-confirmed OP frequently coexists with adductor pathology, and symptoms medially may align more directly with the adductor lesion [20]. Together, these data make it difficult to defend isolated image-based treatments.

Conservative management is the default because direct comparative evidence favoring escalation is sparse. However, “conservative” should not mean rest, modalities, and waiting. It should mean a supervised graded-exposure experiment. The program first reduces the load–capacity mismatch, then progressively recreates the mechanical demands that provoke symptoms. A 12-week reassessment may be used pragmatically to audit diagnosis, adherence, load, and trajectory, but it is not a validated treatment-failure threshold, as time to full return is often longer than this, especially in longstanding cases [33,38]. The relevant question is whether capacity and sport tolerance are improving, not whether an arbitrary number of weeks has passed. Pain monitoring, next-day response, strength symmetry, and field-progression criteria remain unvalidated OP-specific proposals.

The evidence for the intermediate tier remains the weakest part of the pathway. Corticosteroid injection may provide short-term relief, ESWT may accelerate recovery when combined with exercise, and orthobiological procedures are increasingly available. Yet the literature rarely distinguishes treatment of the symphysis from treatment of the adductor origin or the prepubic complex, and co-interventions for rehabilitation are inconsistently reported [37,40,41,42,43,46,47]. A procedure should therefore be framed as a hypothesis test with a defined target and follow-up decision, not as a generic next step. Diagnostic local-anesthetic injection, therapeutic intra-symphyseal corticosteroid injection, and treatment of an adductor or prepubic aponeurotic lesion should therefore be considered separately.

Surgery can be effective in carefully selected refractory cases, but treatment selection remains vulnerable to referral and publication bias. Recent endoscopic series have improved technical and midterm outcome information [54,55], but they still represent only a few dozen highly selected patients. Return-to-sport percentages vary depending on whether athletes who do not attempt return are included in the denominator. Future studies should report return to participation, return to the preinjury sport, return to performance, time, workload, recurrence, complications, and patient-reported outcomes separately. In the broader surgical review, only 30 athletes contributed isolated pubic-symphysis outcomes (23/30 returned to the preinjury level) [50].

The clinician must also avoid two opposite errors. The first is under-treatment: repeatedly asking an athlete with a well-defined, persistent lesion and verified rehabilitation failure to “do more therapy” without changing the diagnostic or therapeutic hypothesis. The second is premature escalation based on an impressive MRI. The evidence-informed decision framework outlined in this review addresses both by requiring reassessment at each transition and by making concordance—not image severity or time alone—the central criterion. The framework is author-proposed and should not be interpreted as a validated algorithm.

## 12. Strengths and Limitations

This review integrates consensus terminology, clinical reliability studies, contemporary anatomy, symptomatic and asymptomatic imaging cohorts, rehabilitation research, procedural evidence, and surgical series through August 2026. It explicitly separates clinical entities from imaging phenotypes and separates direct from indirect evidence and translates it into proposed progression and escalation criteria. The decision framework is intended to be usable in multidisciplinary sports practice and to expose, rather than conceal, uncertainty. Its additional contribution is the explicit distinction between diagnostic and therapeutic injections and between tissue-sparing procedures and stabilization.

The review is narrative and was not conducted under a prospectively registered protocol, duplicate screening, or meta-analysis. Screening was performed by one author with consensus review of uncertain cases; design-specific methodological domains were applied to principal decision-informing studies but do not constitute a full systematic risk-of-bias review. Relevant articles outside PubMed/MEDLINE or in languages other than English may have been missed. Much of the treatment literature consists of small male football cohorts, retrospective studies, case series, and mixed diagnoses. Definitions of OP, pubic-related pain, treatment failure, and return to sport differ substantially, preventing reliable cross-study comparisons. The multiple practical recommendations presented in this review are therefore clinical proposals rather than validated thresholds.

## 13. Future Directions

Future work needs to begin with a minimum diagnostic dataset: standardized Doha-based examination, a pain map, skeletal maturity and pubic-apophyseal status, training exposure, coexisting clinical entities, imaging protocol, anatomical lesion definitions and baseline HAGOS. Studies should recruit women, adolescents, non-football athletes, and nonelite participants rather than extrapolating from adult male footballers. Pubic apophysitis and pubic bone stress injury should be studied separately from adult OP.

Rehabilitation trials should compare clearly described, dose-matched programs and report adherence and sporting load. Prognostic studies are needed to determine whether specific MRI patterns—including IPL lesions, adductor-aponeurotic injury, or marrow edema distribution—predict response rather than merely coexist with symptoms. Injection trials should specify the anatomical target, injectate composition, guidance method, rehabilitation co-intervention, and the purpose beforehand. Comparative surgical registries and, where feasible, pragmatic trials should use consistent indications and include nonoperative comparators.

A core outcome set should distinguish pain reduction from restored performance. At minimum, studies should report HAGOS or an equivalent validated score; return to participation, sport, and performance; time and exposure; recurrence; complications; and patient satisfaction. An agreement concerning what constitutes a high-quality rehabilitation trial and true nonresponse would be more valuable than another isolated technique report.

## 14. Conclusions

In this review, osteitis pubis is retained as an author-proposed clinico-radiological phenotype as a possible clinico-radiological phenotype within pubic-related groin pain, not as an established diagnostic standard or a diagnosis created by imaging alone. Recognizable pubic pain and tenderness, methodical classification of coexisting entities, and careful exclusion of consequential alternatives form the diagnostic foundation. Radiographs and MRI refine this assessment, but common asymptomatic findings require restraint. Pubic bone stress injury, pubic apophysitis, adductor-related pain, and rectus–adductor aponeurotic injury require separate consideration.

Active, supervised, criteria-based rehabilitation with graded restoration of sport load remains first-line treatment. ESWT may be a useful adjunct, while injections and orthobiological procedures should be targeted, purpose-defined, and presented as evidence-limited. Surgical treatment is reasonable for a small subgroup with persistent disability after a verified rehabilitation trial and a concordant pain generator. Endoscopic techniques are promising, but contemporary evidence remains based on small uncontrolled series. The most important next step is not proliferation of treatments; it is standardization of diagnosis, rehabilitation quality, failure criteria, and outcomes. Pain, strength, field-progression, reassessment, and return-to-sport thresholds in the proposed framework remain unvalidated.

## Figures and Tables

**Table 1:** Clinical classification and differential diagnosis of anterior groin pain in athletes. Entries combine consensus definitions, published evidence, and proposed clinical cautions; they are not validated decision rules.

Entity	Typical History and Examination	Imaging Role	Frequent Pitfall
Pubic-related groin pain/symptomatic OP phenotype	Central or paramedian pubic pain aggravated by running, cutting, kicking, rolling in bed, or single-leg loading; recognizable pain on pubic palpation	MRI may show marrow edema, symphyseal irregularity, aponeurotic or ligamentous injury; radiographs may show chronic adaptation	Diagnosing from edema, sclerosis, or lucency without clinical concordance
Adductor-related groin pain	Medial groin pain; tenderness at the adductor origin and pain on resisted adduction	MRI or ultrasound can define acute or chronic myotendinous/entheseal lesions	Assuming all pain near the pubis arises from the joint
Inguinal-related groin pain	Inguinal canal pain/tenderness, aggravated by abdominal resistance, cough, or Valsalva, without a palpable hernia	Dynamic ultrasound or MRI may support selected diagnoses; imaging is not uniformly diagnostic	Using “sports hernia” as a catch-all diagnosis
Iliopsoas-related groin pain	Anterior groin pain with iliopsoas tenderness; pain on resisted hip flexion and/or hip-flexor stretch	MRI/ultrasound may identify bursitis or tendon disease	Failing to distinguish from intra-articular hip pain
Hip-related groin pain	Deep groin pain, mechanical symptoms, restricted motion; FADIR/FABER used as screening rather than definitive tests	AP pelvis and Dunn/lateral radiographs first; MRI/MR arthrography when clinically indicated	Treating cam morphology alone or overlooking coexistence with pubic pain
Bone stress injury/apophyseal injury	Focal bone pain, recent load increase, adolescent growth, pain with impact; may progress to pain at rest	MRI is preferred when radiographs are normal	Continuing sport because early radiographs are negative
Infection, inflammatory disease, visceral or referred pain	Fever, systemic symptoms, night/rest pain, recent pelvic surgery or infection, neurologic, urinary, gynecologic, or abdominal symptoms	Laboratory tests and targeted imaging according to suspected disease	Applying a sports diagnosis before excluding red flags

Abbreviations: FADIR, flexion–adduction–internal rotation; FABER, flexion–abduction–external rotation; MRI, magnetic resonance imaging; OP, osteitis pubis. Pubic-related and adductor-related definitions follow consensus terminology [2]; other entries synthesize evidence and author interpretation.

**Table 2:** Imaging modalities and their influence on clinical decisions. The table describes evidence-informed uses and limitations; it is not a validated imaging algorithm.

Modality	Main Strengths	Important Limitations	Appropriate Decision Influence
AP pelvis ± Dunn/lateral radiographs	Bone morphology, chronic pubic changes, avulsion, hip morphology; inexpensive	Poor sensitivity for early stress injury; pubic changes common in asymptomatic footballers	Exclude gross bone disease and frame hip/pelvic morphology; do not define symptomatic OP alone
MRI pelvis	Bone stress, apophyseal, adductor, aponeurotic, ligamentous, hip, and alternative pathology in one study	Variable protocols and definitions; abnormalities may be incidental; normal scan does not exclude clinical pain	Confirm or refine a clinically generated differential, stage tissue involvement, identify competing targets
Ultrasound	Dynamic inguinal assessment, superficial tendons, low cost, guidance for injection	Operator dependence; limited evaluation of marrow and deep pelvis	Answer a focused tendon/inguinal question or guide a targeted procedure
CT	Detailed osseous anatomy, erosions, cysts, surgical planning	Ionizing radiation and limited soft-tissue contrast	Reserve for unresolved bony questions or operative planning
Image-guided anesthetic injection	Can test immediate task-related response at a suspected site	Spread and placebo effects; no universally accepted response threshold	Use as one component of concordance, not as an independent diagnostic gold standard

**Table 3:** Structured comparison of operations reported for refractory pubic-region pain.

Procedure	Potential Clinical Setting	Stability/Structures	Evidence Signal	Main Risks and Limitations
Open curettage/debridement	Focal, concordant symphyseal pain with a stable pelvic ring after documented rehabilitation nonresponse	Removes degenerative fibrocartilage/subchondral tissue while minimizing ligament disruption	Radic: 16/23 returned to full activity; mean 5.6 months; one late fusion [51]	Persistent pain, incomplete debridement, iatrogenic instability, bladder/neurovascular injury; uncontrolled series
Limited/arthroscope-assisted curettage	Same stable-ring phenotype when a tissue-sparing approach is feasible	Small anterior capsular window; preserves adjacent ligaments	Four of four competitive athletes returned in one case series [58]	Very small cohort; technical dependence; no comparator
Endoscopic symphysectomy	Selected refractory cases with concordant symphyseal target, usually without established pelvic-ring instability	Central/anterior resection with intended preservation of posterior/deep stabilizers	10-player and 14-patient level-IV series reported improvement; overall RTS 9/12 sport participants in the 2026 cohort [54,55]	Scrotal swelling, fluid extravasation, bladder/neurovascular injury, under- or over-resection, postoperative instability; no comparative evidence
Wedge resection	Historically used for refractory pain when joint resection is intended but ligament preservation is possible	Wedge removes diseased joint while retaining anterior/inferior stabilizing structures	Small, heterogeneous historical series [49]	Posterior pelvic pain/instability may appear late; evidence largely non-athletic and outdated
Arthrodesis	Documented symptomatic instability, painful pelvic-ring instability, or salvage after failed resection/curettage	Requires assessment of anterior and posterior ring; fusion with fixation and usually graft	Williams: 7/7 rugby players returned after fusion; historical >2-mm criterion was cohort-specific [22]	Nonunion, hardware irritation/removal, graft morbidity, altered pelvic mechanics, longer rehabilitation
Adductor/aponeurotic, inguinal, or hip procedure	Only when the associated lesion is independently clinically dominant and concordant	Target is outside or in addition to the symphysis; combined surgery may be appropriate for two established diagnoses	Outcomes cannot be attributed to the symphysis when procedures are combined [11,50,56,57]	Overtreatment, increased morbidity, and inability to determine which component produced benefit

## Data Availability

No new data were created or analyzed in this study. Data sharing is not applicable to this article.

## Supplementary Materials

The following supporting information can be downloaded at https://www.mdpi.com/article/10.3390/jcm15187187/s1; Supplementary Material provides the complete PubMed/MEDLINE search syntax and record-selection summary; Table S1 reports the actual diagnostic population, sample size, comparator, follow-up, principal quantitative findings, complications, risk-of-bias concerns, and directness of evidence for the principal studies.
