# Supplementary material for: Osteitis Pubis in Athletes in the Era of Pubic-Related Groin Pain: Contemporary Concepts in Diagnosis, Rehabilitation, and Surgical Decision-Making
Source: J Clin Med. 2026 Sep 16;15(18):7187. doi: 10.3390/jcm15187187 (PMC13607891; doi:10.3390/jcm15187187)
Supplement: Supplementary file 1 [file jcm-15-07187-s001.zip › jcm-4551116-supplementary.pdf]

Supplementary material

Osteitis Pubis in Athletes in the Era of Pubic-Related Groin Pain: Contemporary Concepts in Diagnosis, Rehabilitation, and Surgical Decision-Making

PubMed/MEDLINE Search Strategy

Database: PubMed/MEDLINE. Search performed: August 2026. Publication-date cutoff: 4 August 2026. Language filter: none.

MeSH note using Title/Abstract terms.

(("osteitis pubis"[Title/Abstract] OR "pubic-related groin pain"[Title/Abstract] OR "pubic related groin pain"[Title/Abstract] OR "pubic bone stress"[Title/Abstract] OR "pubic symphysis"[Title/Abstract] OR "athletic pubalgia"[Title/Abstract] OR "core muscle injury"[Title/Abstract] OR "prepubic aponeurotic complex"[Title/Abstract] OR "secondary cleft sign"[Title/Abstract]) AND (athlet*[Title/Abstract] OR sport*[Title/Abstract] OR football*[Title/Abstract] OR soccer[Title/Abstract] OR hockey[Title/Abstract] OR rugby[Title/Abstract] OR skater*[Title/Abstract])) AND ("1900/01/01"[Date - Publication] : "2026/08/04"[Date - Publication])

The query shown above uses a publication-date cutoff of 4 August 2026. A contemporaneous retrieval count for that search is not available.

Record-Selection Summary

Study selection for this narrative review was iterative and was not documented in a contemporaneous screening log. The original retrieval count, intermediate screening counts, and the numbers selected separately from database and citation searching cannot be verified and are not reported. The revised manuscript cites 57 scientific or clinical sources and the WADA Prohibited List, giving 58 references in total. The cited-source count is not a systematic-review inclusion total.

Selection stage	Number / note
PubMed/MEDLINE records identified	Not verifiable from contemporaneous records; not reported
Records excluded after title/abstract screening	Not recorded contemporaneously; not reported
Full-text reports assessed	Not recorded contemporaneously; not reported
Publications included from database search	Not separately documented; not reported
Additional publications identified through citation searching	Not separately documented; not reported
Scientific or clinical sources cited in the manuscript	57 (reference count)
Regulatory source cited separately	2026 WADA Prohibited List (not counted as clinical evidence)

Screening and extraction were performed by R.G.V. Uncertain eligibility, diagnostic-population classification, and interpretation were discussed with M.G. and A.M. until consensus was reached. No language filter was applied at search; reports without sufficient English-accessible information for reliable extraction were excluded, creating a possible language bias.

Table S1. Study-Level Evidence Informing Diagnosis and Management

Panel A. Principal studies informing diagnosis, rehabilitation, injections, and evidence interpretation

Study	Actual diagnostic population / design	N / comparator	Follow-up	Principal quantitative findings	Complications	Principal methodological limitation / qualitative bias concern	Directness for OP
Hölmich 1999 [S1]	Longstanding adductor-related groin pain; randomized trial	68 randomized; active training vs passive physiotherapy	4 months	Pain-free return to sport among assessed athletes: 23/29 (79%) vs 4/30 (13%)	Not a principal reported outcome	Unblinded exercise comparison, incomplete outcome data; population was adductor-related, not isolated OP	Indirect
O'Connell 2002 [S2]	Athletes with clinically and radiographically diagnosed OP; symphyseal-cleft injection series	16; no comparator	Immediate, 2 weeks, 2 and 6 months	14/16 partially improved and resumed sport within 48 h; 10/16 retained relief at 2 weeks; 5/16 were symptom-free at 2 and 6 months	No target-specific complication rate reported; 1 required further injections and 1 was referred for fusion	Small uncontrolled cohort; diagnostic anesthetic and therapeutic corticosteroid effects cannot be separated	Direct, low certainty
Verrall 2007 [S3]	Professional Australian footballers with chronic groin injury diagnosed as pubic bone stress injury; case series	27; no comparator	Two playing seasons	89% returned during the next season and 100% by the second; symptom-free: 41% at the start and 67% at the end of the next season	Not a principal reported outcome	No comparator; rest-dominant protocol; diagnostic construct differs from contemporary clinical OP	Partially direct: bone-stress phenotype
Choi 2011 [S4]	Athletic OP and pubic osteomyelitis treatment literature; systematic review	195 athletes across low-level reports	Heterogeneous	Approximately 59% of injected athletes returned to sport; treatment estimates were heterogeneous	Inconsistently reported	All included treatment evidence was level IV; heterogeneous populations, interventions, and outcome definitions	Direct topic, low certainty
Sailly 2015 [S5]	Adolescent footballers with adductor-related pain and an open pubic apophysis; imaging case series	26 symptomatic; 31 asymptomatic CT controls	Cross-sectional	Stress-related apophyseal abnormalities were identified in symptomatic adolescents; maturation may extend into the early twenties	Not applicable	Retrospective imaging-selected series; controls underwent CT for different indications	Differential diagnosis, not adult OP
Schöberl 2017 [S6]	Footballers with pubic overload/aseptic OP; randomized ESWT comparison plus separate rest-treated control	44 randomized: ESWT+rehab 26 vs sham+rehab 18; separate nonrandomized rest group 51	1 year	42/44 randomized participants returned within 4 months without recurrence; the rest group returned later and 26/51 recurred	No major treatment complication emphasized	Randomization applied only to ESWT versus sham; the rest group was not a third randomized arm	Partially direct: mixed pubic-overload construct
McAleer 2017 [S7]	Professional and academy soccer players with MRI-confirmed pubic bone stress/OP; criteria-based rehabilitation case series	5; no comparator	Mean final follow-up 29.6 months (16–33)	Mean return to training 40.6 days (30–60); mean return to play 49.4 days (38–72); no recurrence	None reported	Five cases, no comparator; progression criteria were not prospectively validated	Partially direct, very low certainty
Hatem 2021 [S8]	Surgery for inguinal-, pubic-, and adductor-related chronic groin pain; systematic review	47 studies / 2737 total; only 30 isolated pubic procedures	>6 months in included studies	Return to preinjury level for isolated pubic procedures: 7/7 arthrodesis and 16/23 curettage (23/30)	Pubic-specific complications incompletely reported	44/47 studies were level IV; marked clinical and procedural heterogeneity	Direct only for the 30-patient pubic subgroup
Eberbach 2021 [S9]	Professional academy soccer players with longstanding clinically defined pubic-related groin pain; prospective cohort	14; no comparator	24 months	14/14 returned; mean 135.3 ± 83.9 days; recurrence 1/14	No major complication	Small uncontrolled youth cohort; program effect cannot be separated from natural history	Direct: pubic-related clinical population
Mahmoud 2024 [S10]	Adolescent male footballers labeled as OP; comparative rehabilitation study	50; modified active 24 vs traditional 26	12 weeks	Both groups improved; modified program favored strength/function and traditional program favored pain outcomes	No major event reported	Short follow-up, single setting, and limited reporting of allocation/blinding	Direct by study label; adolescent-specific
Hynes 2023 [S11]	Athletes referred for groin MRI; cross-sectional cohort	470; no asymptomatic comparator	Cross-sectional	119/470 MRI examinations (25.3%) were normal; multiple coexisting injury patterns were observed	Not applicable	Referral and verification bias; clinical reference standard was limited	Indirect diagnostic context
Mathieu 2025 [S12]	Clinically defined pubic-related groin pain versus adductor-related groin pain control group; cross-sectional MRI	60 pubic-related (56 M/4 F) + 28 controls (19 M/9 F)	Cross-sectional	IPL lesion 20/60 (33.3%; definite or equivocal) versus 0/28 controls; trauma OR 8.76 (95% CI 2.56–29.91)	Not applicable	Cross-sectional study; no outcome-based validation; equivocal MRI findings included in the 33.3% estimate	Direct diagnostic evidence
Nielsen 2025 [S13]	Symptomatic male footballers, asymptomatic male footballers, and asymptomatic male non-football athletes; radiographic cohort	39 / 18 / 20	Cross-sectional	Lucency 87%/83%/40%; proliferation 67%/61%/25%; sclerosis 64%/50%/15%; symptomatic vs asymptomatic footballers $p \geq 0.39$	Not applicable	Cross-sectional male cohort; no causal or prognostic inference	Direct evidence of limited imaging specificity
Eraslan 2025 [S14]	Male athletes selected by MRI-defined OP; retrospective cohort	132; injection subgroups nonrandom	Retrospective	Adductor lesion in 90%; symptoms were better aligned with the adductor lesion side than with bilateral OP; crude injection-group RTS differences were nonsignificant	Inconsistently reported	Imaging-selected retrospective cohort; substantial confounding by indication	Direct label, mixed pain generators
Sato 2026 [S15]	Asymptomatic elite youth soccer players versus asymptomatic non-athletes; MRI study	40 vs 32	Cross-sectional	Subapophyseal edema 77.5% vs 18.8%; cortical irregularity 82.5% vs 25.0%	Not applicable	Cross-sectional male youth sample; control MRI indication differed	Differential diagnosis / imaging specificity
Presutti 2026 [S16]	Athletic bone stress injury; clinical narrative review	Not applicable	Not applicable	Synthesizes MRI-based diagnosis, load modification, RED-S assessment, and criteria-based return	Not applicable	Narrative review; no formal evidence grading	Indirect: bone-stress differential

Panel B. Cohort-level surgical evidence

This panel includes the athlete-focused pubic-symphyseal cohorts cited or discussed in the manuscript, additional direct cohorts identified during verification, and selected adjacent or combined-procedure cohorts needed to show indirectness. Isolated case reports, predominantly non-athletic cohorts, and technique descriptions without an outcome cohort are not presented as cohort evidence. NR, not reported in the accessible primary report or abstract.

Study / design	Diagnostic population; sex; symptom duration	N / comparator	Prior nonoperative treatment / diagnostic injection	Instability assessment	Procedure / concomitant procedure	Follow-up	Return-to-sport or principal outcome	Complications / reoperations	Directness / principal limitation
Grace 1989 [S17] / case series	Recalcitrant OP; sex and sport NR; mean symptoms 32 months	10; no comparator	≥6 months; diagnostic injection NR	Dynamic and posterior-ring assessment NR	Open wedge resection	Mean 14 months early; 92 months long term	All markedly improved and fully active early; 3/10 dissatisfied long term; preinjury-performance definition NR	1 later bilateral sacroiliac arthrodesis for instability-related pain	Partially direct; historical mixed population, small uncontrolled cohort
Williams 2000 [S18] / case series, level IV	Male rugby players with OP and >2-mm vertical translation; exact symptom duration NR	7; no comparator	≥13 months; diagnostic injection NR	>2-mm translation on Flamingo views; posterior ring NR	Arthrodesis with bone graft and compression plate	Mean 52.4 months	7/7 symptom-free and returned to rugby; mean reported RTS 6.6 months	Hemospermia 1; intermittent scrotal swelling 1; no residual symphyseal instability	Direct instability subtype; very small selected cohort; cohort-specific threshold
Mulhall 2002 [S19] / two-patient series	Male professional soccer players, ages 25 and 26; symptoms 1 and 1.5 years	2; no comparator	Refractory to conservative care; diagnostic injection NR	Dynamic and posterior-ring assessment NR	Open symphyseal curettage; local methylprednisolone 40 mg plus 0.5% bupivacaine reported	NR	2/2 symptom-free and returned within 6 months	No complication reported	Direct; two patients; detailed data partly available through later evidence synthesis
Paajanen 2005 [S20] / case series	Elite male athletes with chronic OP: 3 soccer, 1 bandy, 1 cross-country skier; symptoms several months	5; no comparator	Conservative care failed; diagnostic injection NR	Dynamic and posterior-ring assessment NR	Video-assisted 10×15-cm polypropylene mesh in preperitoneal retropubic space	12 months	5/5 returned at 1–2 months; no pubic tenderness or pain at 1 year	No complication reported	Direct by historical OP criteria; very small uncontrolled series
Paajanen 2008 [S21] / case-control, level IV	Athletes with severe pubic enthesopathy/OP; sex and symptom duration NR	8 surgery; 8 nonoperative; 20 asymptomatic imaging controls	Nonoperative comparator: physiotherapy and corticosteroids; diagnostic injection NR	Dynamic and posterior-ring assessment NR	Totally extraperitoneal retropubic mesh	Mean 2.7 years (1–6)	7/8 surgical athletes returned after 2 months; 4/8 nonoperative athletes had disabling symptoms at 1 year	No surgical complication reported	Partially direct; nonrandom allocation and baseline severity imbalance
Radic 2008 [S22] / retrospective case series, level IV	Treatment-resistant OP; 22 M/1 F; mean symptoms 13.22 months	23; no comparator	Initial nonoperative treatment failed; diagnostic injection NR	Dynamic and posterior-ring criteria NR	Isolated open symphyseal curettage	Mean 24.31 months (12.5–59.6)	21/23 pain-free running at 3.14 months; 17/23 training at 4.44; 16/23 full activity at 5.63	No significant perioperative complication; 1 later fusion for persistent symptoms	Direct; retrospective, selected, uncontrolled
Hechtman 2010 [S23] / case series, level IV	Male football athletes (2 professional, 2 collegiate); mean symptoms 10 months (6–13)	4; no comparator	Rest, rehabilitation and CT-guided steroid injection; ≥6 months	Formal dynamic criteria NR; ligament-sparing intent	Arthroscope-assisted limited open curettage through small anterior capsular incision	Mean 50 months (47–52)	4/4 returned to same level; mean RTS 3 months (2–8)	No complication or reoperation	Direct; four-patient uncontrolled series
Hopp 2013 [S24] / prospective case series	Competitive nonprofessional soccer players with degenerative OP plus adductor-longus lesion; sex NR; ≥12 months failed care	5; no comparator	≥12 months; arthrography identified adductor microlesions; anesthetic injection NR	Formal preoperative dynamic threshold NR; no postoperative instability	Arthroscopic symphyseal curettage plus adductor debridement and suture-anchor reattachment	Mean 18.2 months	5/5 returned to full activity; mean 14.4 weeks	1 corona-mortis bleeding/vascular injury requiring revision/ligation	Partially direct; combined pubic and adductor procedure, small uncontrolled cohort
Matsuda 2015 [S25] / multicenter retrospective case series, level IV	Adult athletes with symptomatic FAIS and associated OP; 4 M/3 F; symptom duration NR	7; no comparator	Prior care and diagnostic injection incompletely reported	No anterior or posterior ring instability pre- or postoperatively	Endoscopic symphysectomy combined with FAIS surgery	Mean 2.9 years (2–5)	RTS definition/denominator NR; VAS 6.7→1.5; NAHS 50.2→84.7	Transient scrotal swelling 2; arthrodesis for persistent pain 1	Indirect combined hip–pubic procedure; treatment effect cannot be isolated
Zimmerer 2022 [S26] / case series	Athletes with pubic-related pain/OP and FAIS; 7 M/3 F; symptom duration NR	10; no comparator	Refractory; detailed prior treatment and diagnostic injection NR	Formal dynamic and posterior-ring criteria NR	Arthroscopically assisted minimally invasive symphysioplasty plus FAIS treatment	Mean 5.1 years (2–9)	10/10 returned to previous sport level; NAHS 64.4±15.1→91.4±9.8	Not reported in abstract	Indirect combined procedure; small uncontrolled cohort
Emblom 2018 [S27] / retrospective case series, level IV	Rectus–adductor aponeurotic plate injury; 73 M/12 F among followed athletes; symptom duration NR	100 eligible; 85 followed	Failed 6–12 weeks; local steroid injections in some; not a standardized diagnostic test	Not a symphyseal-instability cohort	Adductor longus fractional lengthening/limited tenotomy plus adductor-to-rectus turn-up flap; heterogeneous prior/additional procedures	Mean 25.8±13.2 months (6.9–53.8)	82/85 (96%) returned; mean 4.1±2.3 months	6/85 (7%): 2 suspected retears, 2 superficial infections, 1 hematoma, 1 heterotopic-ossification reoperation; 8 transient sexual dysfunction	Indirect adjacent aponeurotic lesion; retrospective, uncontrolled, heterogeneous procedures
Saito 2019 [S28] / retrospective case series, level IV	Competitive soccer players with symptomatic FAIS; OP on radiographs 67.8%, pubic BME 35.7%; sex/duration NR in abstract	28 hips; no comparator	Prior care and diagnostic injection NR	Symphyseal instability was not the operative target	Hip arthroscopy for FAIS; no direct pubic procedure	Minimum 2 years	92% returned to same/higher soccer level; median 5.5 months	Not a principal abstract outcome	Indirect hip-related population; no pubic procedure effect
Ganal 2023 [S29] / case series, level IV	Male competitive soccer players with isolated recalcitrant OP; mean symptoms 20.5±16.5 months	10; no comparator	Rest, rehabilitation, NSAIDs, pubic corticosteroid injection, activity modification; ≥12 months	AP/Flamingo >2 mm excluded; no postoperative instability	Isolated arthroscopic symphysiectomy/debridement	Mean 72.2±13.4 months (60–97)	10/10 returned; mean 4.6±1.7 months; VAS 9.3→0.2	Transient scrotal/testicular swelling 4, resolved; no recurrence or reoperation	Direct; small selected single-center uncontrolled cohort
Quesada-Jimenez 2026 [S30] / retrospective case series, level IV	Refractory OP; sex and symptom duration not reported in abstract	14/15 followed; no comparator	Failed nonoperative care; diagnostic injection NR	Detailed dynamic and posterior-ring criteria NR in abstract	Endoscopic pubic symphysectomy; concomitant procedures incompletely reported in abstract	Minimum 2 years	12 preoperative sport participants; 9/12 returned within 1 year; 9/9 who attempted returned; 3 did not attempt	Not reported in the abstract; rare events cannot be estimated reliably	Direct; small uncontrolled cohort; denominator-sensitive RTS

Supplementary References

S1. Hölmich P, Uhrskou P, Ulnits L, et al. Effectiveness of active physical training as treatment for long-standing adductor-related groin pain in athletes: randomised trial. Lancet. 1999;353:439–443. doi:10.1016/S0140-6736(98)03340-6.
S2. O'Connell MJ, Powell T, McCaffrey NM, O'Connell D, Eustace SJ. Symphyseal cleft injection in the diagnosis and treatment of osteitis pubis in athletes. AJR Am J Roentgenol. 2002;179:955–959. doi:10.2214/ajr.179.4.1790955.
S3. Verrall GM, Slavotinek JP, Fon GT, Barnes PG. Outcome of conservative management of athletic chronic groin injury diagnosed as pubic bone stress injury. Am J Sports Med. 2007;35:467–474. doi:10.1177/0363546506295180.
S4. Choi H, McCartney M, Best TM. Treatment of osteitis pubis and osteomyelitis of the pubic symphysis in athletes: a systematic review. Br J Sports Med. 2011;45:57–64. doi:10.1136/bjsm.2008.050989.
S5. Sailly M, Whiteley R, Read JW, Giuffre B, Johnson A, Hölmich P. Pubic apophysitis: a previously undescribed clinical entity of groin pain in athletes. Br J Sports Med. 2015;49:828–834. doi:10.1136/bjsports-2014-094436.
S6. Schöberl M, Prantl L, Loose O, et al. Non-surgical treatment of pubic overload and groin pain in amateur football players: a prospective double-blinded randomised controlled study. Knee Surg Sports Traumatol Arthrosc. 2017;25:1958–1966. doi:10.1007/s00167-017-4423-z.
S7. McAleer SS, Lippie E, Norman D, Riepenhof H. Nonoperative management, rehabilitation, and functional and clinical progression of osteitis pubis/pubic bone stress in professional soccer players: a case series. J Orthop Sports Phys Ther. 2017;47:683–690. doi:10.2519/jospt.2017.7314.
S8. Hatem M, Martin RL, Bharam S, et al. Surgical outcomes of inguinal-, pubic-, and adductor-related chronic pain in athletes: a systematic review based on surgical technique. Orthop J Sports Med. 2021;9:23259671211023116. doi:10.1177/23259671211023116.
S9. Eberbach H, Fürst-Meroth D, Kloos F, et al. Long-standing pubic-related groin pain in professional academy soccer players: a prospective cohort study on possible risk factors, rehabilitation and return to play. BMC Musculoskelet Disord. 2021;22:958. doi:10.1186/s12891-021-04837-x.
S10. Mahmoud WS, Ibrahim MM, Radwan NL. Efficacy of modified active physical therapy program on pain, muscle strength, and function in adolescent football players with osteitis pubis. Bull Fac Phys Ther. 2024;29:2. doi:10.1186/s43161-023-00170-7.
S11. Hynes JP, O'Flaherty M, Glynn D, Eustace S, Kavanagh EC. Imaging of groin pain in athletes: patterns of injury at MRI and gender differences therein. Ir J Med Sci. 2023;192:1411–1418. doi:10.1007/s11845-022-03126-3.
S12. Mathieu T, Van Glabbeek F, Van Nassauw L, Denteneer L, Bouziotis J, Van Dyck P. Inferior pubic ligament injury: magnetic resonance imaging assessment in athletes with pubic-related groin pain. Orthop J Sports Med. 2025;13:23259671251380879. doi:10.1177/23259671251380879.
S13. Nielsen MF, Hölmich P, Branci S, et al. Pubic-related radiographic findings in male football players with long-standing groin pain, and asymptomatic controls—are they clinically relevant? Scand J Med Sci Sports. 2025;35:e70068. doi:10.1111/sms.70068.
S14. Eraslan A, Kose O. Prevalence and patterns of adductor lesions on MRI in athletes with osteitis pubis. J Orthop Traumatol. 2025;26:65. doi:10.1186/s10195-025-00875-6.
S15. Sato VN, Moriwaki TL, do Amaral e Castro A, Skaf AY, Yamada AF. Pubic apophyseal MRI findings in asymptomatic elite youth soccer players: a cross-sectional comparison with non-athletes. Skeletal Radiol. 2026. doi:10.1007/s00256-026-05329-6.
S16. Presutti F, Paoletti S, Conte F, et al. Stress fracture in athletes: a practical approach. J Clin Med. 2026;15:3077. doi:10.3390/jcm15083077.
S17. Grace JN, Sim FH, Shives TC, Coventry MB. Wedge resection of the symphysis pubis for the treatment of osteitis pubis. J Bone Joint Surg Am. 1989;71:358–364. doi:10.2106/00004623-198971030-00008.
S18. Williams PR, Thomas DP, Downes EM. Osteitis pubis and instability of the pubic symphysis: when nonoperative measures fail. Am J Sports Med. 2000;28:350–355. doi:10.1177/03635465000280031101.
S19. Mulhall KJ, McKenna J, Walsh A, McCormack D. Osteitis pubis in professional soccer players: a report of outcome with symphyseal curettage in cases refractory to conservative management. Clin J Sport Med. 2002;12:179–181. doi:10.1097/00042752-200205000-00006.
S20. Paajanen H, Heikkinen J, Hermunen H, Airo I. Successful treatment of osteitis pubis by using totally extraperitoneal endoscopic technique. Int J Sports Med. 2005;26:303–306. doi:10.1055/s-2004-820975.
S21. Paajanen H, Hermunen H, Karonen J. Pubic magnetic resonance imaging findings in surgically and conservatively treated athletes with osteitis pubis compared to asymptomatic athletes during heavy training. Am J Sports Med. 2008;36:117–121. doi:10.1177/0363546507305454.
S22. Radic R, Annear P. Use of pubic symphysis curettage for treatment-resistant osteitis pubis in athletes. Am J Sports Med. 2008;36:122–128. doi:10.1177/0363546507306160.
S23. Hechtman KS, Zvijac JE, Popkin CA, Zych GA, Botto-van Bemden A. A minimally disruptive surgical technique for the treatment of osteitis pubis in athletes. Sports Health. 2010;2:211–215. doi:10.1177/1941738110366203.
S24. Hopp SJ, Culemann U, Kelm J, Pohlemann T, Pizanis A. Osteitis pubis and adductor tendinopathy in athletes: a novel arthroscopic pubic symphysis curettage and adductor reattachment. Arch Orthop Trauma Surg. 2013;133:1003–1009. doi:10.1007/s00402-013-1777-7.
S25. Matsuda DK, Ribas M, Matsuda NA, Domb BG. Multicenter outcomes of endoscopic pubic symphysectomy for osteitis pubis associated with femoroacetabular impingement. Arthroscopy. 2015;31:1255–1260. doi:10.1016/j.arthro.2015.02.005.
S26. Zimmerer A, Ramirez L, Astarita E, Bellotti V, Cárdenas C, Ribas M. Arthroscopically assisted minimally invasive symphysioplasty for the treatment of pubic-related groin pain. Oper Orthop Traumatol. 2022;34:109–116. doi:10.1007/s00064-021-00753-4.
S27. Emblom BA, Mathis T, Aune K. Athletic pubalgia secondary to rectus abdominis–adductor longus aponeurotic plate injury: diagnosis, management, and operative treatment of 100 competitive athletes. Orthop J Sports Med. 2018;6:2325967118798333. doi:10.1177/2325967118798333.
S28. Saito M, Utsunomiya H, Hatakeyama A, et al. Hip arthroscopic management can improve osteitis pubis and bone marrow edema in competitive soccer players with femoroacetabular impingement. Am J Sports Med. 2019;47:408–419. doi:10.1177/0363546518819099.
S29. Ganal İ, Çelen ZE. Midterm functional outcomes of arthroscopically treated recalcitrant osteitis pubis in competitive soccer players. Orthop J Sports Med. 2023;11:23259671231203677. doi:10.1177/23259671231203677.
S30. Quesada-Jimenez R, Kahana-Rojkind AH, Walsh EG, Domb BG. Endoscopic pubic symphysectomy for refractory osteitis pubis provides positive functional outcomes and high return-to-sport rate at a minimum 2-year follow-up. Arthroscopy. 2026;42:879–885. doi:10.1002/arj.70075.
